# How Can I Trust You? The Effect of Risk and Automation Failures on Trust and Reliance Behavior

**DOI:** 10.1177/00187208251398449

**Published:** 2025-12-02

**Authors:** Nikolai Ebinger, Norah Neuhuber, Bettina Kubicek

**Affiliations:** 1 Virtual Vehicle Research GmbH, Austria; 2 27267 University of Graz, Austria

**Keywords:** conditional driving automation, trust in automation, driving simulator, monitoring, automation use

## Abstract

**Objective:**

We examine how risk and automation failures in conditional driving automation (SAE Level 3) influence drivers’ calibration of trust and reliance behavior in the form of system use and monitoring.

**Background:**

Conditionally automated driving brings a challenging new role for drivers, who are permitted to engage in non-driving-related activities but must take back control in certain situations.

**Methods:**

Participants completed three drives in a driving simulation with conditional driving automation. The first drive was with low risk and the second drive was with high risk implemented in the simulation. The third drive included either early or late automation failure.

**Results:**

Participants reported lower trust, took over manual control more often, and monitored more when driving under high risk than when driving under low risk. After experiencing an automation failure, trust decreased immediately but fully recovered over time. Driver’s monitoring increased and decreased immediately as the failure started and ended. The timing of automation failure did not influence its impact on trust.

**Conclusion:**

The results indicate that drivers respond appropriately to risk. Trust develops dynamically in case of an automation failure, but failure timing does not influence this process. From an applied perspective, drivers would benefit from assistance in re-calibrating trust after automation failure.

**Application:**

Based on our findings, we argue that incorporating drivers’ mental model formation process into the feedback loop of trust and reliance behavior calibration could enhance the theoretical understanding of trust calibration.

Driving automation has the potential to reduce accidents related to human error and thus contribute to the European vision of zero traffic fatalities by 2050 (European Commission, 2011). While full driving automation (SAE Level 5, SAE International, 2021) is not expected soon, in 2022 the first car with conditional driving automation (Level 3) was officially approved and made available for sale (Mercedes-Benz Drive Pilot). Conditional driving automation takes over the drivers’ main tasks for some periods of driving so that drivers can temporarily perform non-driving-related activities (NDRAs). Consequently, the driver’s role changes and the reduction in tasks could reduce human error related accidents.

However, the changed role of the driver is challenging, and instead of reducing accidents, it may introduce new hazards. While drivers can perform NDRAs with conditional driving automation, they must immediately take over control if requested by the system. In addition, drivers must detect “performance-relevant system failures in vehicle systems” (SAE International, 2021, p. 28). The new driver role raises the question of whether drivers can fulfill their role by accurately calibrating their trust and reliance on automation in critical situations, such as those involving high risk and automation failures.

Central aspects of how drivers calibrate trust and reliance are not yet fully understood. Drivers can identify high-risk situations and reduce their trust accordingly (Li et al., 2019), but it remains unclear whether this is reflected in their reliance behavior. Furthermore, when confronted with automation failures, drivers reduce their trust (Kraus et al., 2020), but it is still unclear how they recalibrate trust once the conditional driving automation resumes reliable operation. Finally, it remains debated whether, and in what way, the timing of an automation failure influences the extent of trust reduction. Studies using different methodologies have suggested that either early (Desai et al., 2013) or late (Desai et al., 2012) failures may have stronger effects on trust.

To address these open questions in understanding drivers’ trust and reliance behavior calibration, we conducted a driving simulator study with varying risk and automation failure scenarios. In the study, we simultaneously assessed trust, automation use, and monitoring throughout the driver-automation interaction. A temporally specific trust assessment allowed us to analyze the dynamics of trust calibration and directly evaluate whether the timing of automation failures influences drivers’ trust calibration.

Our research deepens the understanding of how drivers calibrate trust and reliance in conditional driving automation. By focusing on temporal aspects, we show the full range and dynamics of drivers’ trust calibration in a situation with temporary automation failure. Furthermore, we examine whether the timing of automation failure influences drivers’ trust. Based on our findings, we discuss practical applications.

## Trust in Automation and Reliance Behavior

Trust is defined as an “attitude” that involves a “willingness to be vulnerable” (Mayer et al., 1995) to the actions of an automated system in situations “characterized by uncertainty” (Lee & See, 2004). Trust plays a central role in the driver-automation interaction, as it affects automation use (Lee & See, 2004). Within a feedback loop, trust is influenced by the perception of automation performance and in turn informs reliance behavior (Hoff & Bashir, 2015; Lee & See, 2004).

Reliance behavior, sometimes also referred to as trust behavior (e.g., in Mayer et al., 1995), is the behavior that results from the attitude of trust (Hoff & Bashir, 2015; Lee & See, 2004). The specific form of potential reliance behavior is contingent on the particular automation use situation (Mayer et al., 1995). In the context of conditional driving automation (Level 3, SAE International, 2021), the automation can take over lateral and longitudinal control of the vehicle, as well as monitoring of the surrounding environment. Thus, if the driver trusts the automation, they will likely rely on it by having it activated to perform these functions and reducing their monitoring of the driving task.

## Risk, Trust in Automation, and Reliance Behavior

Risk is a central aspect of many trust definitions and is essential for understanding trust in automation (Stuck et al., 2021). ISO 12100:2010 defines risk as the “combination of the probability of occurrence of harm and the severity of that harm” (International Organization for Standardization, 2010). While risk is a continuous phenomenon, ranging from no risk or low risk to very high levels of risk (e.g., Ma et al., 2010), researchers who manipulate risk in driving automation experiments often rely on categorical risk implementations (e.g., Ajenaghughrure et al., 2020; Li et al., 2019).

In the driving context, risk perception was approached by measuring the safety feeling, worrying, estimation of probability, and concern regarding being injured or injuring others in a traffic crash (Ma et al., 2010). In automated driving, high-risk situations include bicycles close by or vehicle swerving (Li et al., 2019). In simulated settings, many studies further use monetary incentives to ensure that some level of risk is present. For example, Dzindolet et al. (2003) used monetary rewards based on correct decisions, while Lee & Moray (1992, 1994) and Desai et al. (2012, 2013) incorporated financial performance-based incentives.

According to Hoff and Bashir’s (2015) trust model, risk influences situational trust. Hoff and Bashir (2015) argue that trust is always linked to uncertainty and that therefore risk plays a crucial role in trust development. The higher the risk, the lower the trust in the automation. In line with this argument, Li et al. (2019) and He et al. (2022) showed that risk reduces drivers’ trust in driving automation. Thus, aiming at replicating this finding, we propose:

*Hypothesis 1a:* Trust in automation is lower when driving in a high risk situation than in a low risk situation.

Optimally, the effect of high risk on trust should be reflected in drivers’ reliance behavior. According to the trust-reliance calibration feedback loop (Hoff & Bashir, 2015; Lee & See, 2004) trust informs reliance behavior. However, trust and reliance behavior are not perfectly aligned, and a mismatch may occur. Existing findings on the trust-reliance relation in the driving context are mixed: several studies found a correlation between trust and monitoring behavior (Hergeth et al., 2016; Körber et al., 2018; Walker et al., 2018), whereas others did not (Gold et al., 2015; Schwarz et al., 2019). This inconsistency is also present in other types of reliance behavior. In their study of decision assistance in the context of risk, Hoesterey & Onnasch (2023) found a mismatch between trust and reliance behavior in the form of verifying automation behavior.

Thus, it is not yet fully understood whether drivers who trust conditional driving automation less in situations of high risk (Li et al., 2019) adapt their reliance behavior by increasing their monitoring of the driving task and taking manual control more often. Previous research on the association between risk and reliance behavior also yielded inconsistent results. In a general decision-making task, higher costs of wrong decisions were associated with less reliance on automated decision suggestions (Ezer et al., 2008). Similarly, in a desk-based driving game where drivers encountered obstacles involving ethical decisions and events such as inflamed cars or even corpses on the street, more extreme situations led drivers to take over manual control more frequently, indicating lower reliance on the automation (Ajenaghughrure et al., 2020). Contrary to these results, Perkins et al. (2010) did not find reduced reliance on navigation suggestions in riskier situations.

In light of these inconsistent empirical findings, we base our hypotheses on the assumed trust-reliance calibration feedback loop (Hoff & Bashir, 2015; Lee & See, 2004) and examine two distinct indicators of reliance behavior in driving: drivers’ monitoring and their actual use of the automation. Therefore, we propose:

*Hypothesis 1b:* Driving automation is used less in a high risk situation than in a low risk situation.
*Hypothesis 1c:* Monitoring is higher when driving in a high risk situation than in a low risk situation.

## Temporary Automation Failures

### Temporary Automation Failures and Trust Calibration

The trust drivers place in automation is inextricably linked to the behavior of the automation, including instances of temporary failures (Lee & See, 2004). According to the trust-reliance calibration feedback loop (Hoff & Bashir, 2015; Lee & See, 2004), drivers are expected to diminish their trust in automation in the event of failures and rebuild their trust when the automation functions reliably again. Consequently, the development of appropriate levels of trust in automation necessitates the calibration and alignment of trust with the capabilities of the automation at any given moment. This sensitivity to changes in automation capabilities over time is referred to as temporal specificity, which represents a key criterion for trust appropriateness (Lee & See, 2004).

In the context of temporal specificity, it is important to understand the temporal specifics of trust calibration in the context of automation failures. Although it is assumed that there is a negative correlation between the occurrence of failures and trust (Khastgir et al., 2017), and that drivers adapt their trust across driving situations (Zhang et al., 2025), the dynamics of trust in the context of temporary failures have yet to be elaborated upon. The trust-reliance calibration feedback loop (Hoff & Bashir, 2015; Lee & See, 2004) describes a continuous adaptation process; however, it does not specify the duration of the decrease in trust after an automation failure. Furthermore, the feedback loop does not elucidate the temporal dynamics of the trust rebuilding process, including its onset and duration once the automation functions reliably again. According to Khastgir et al. (2017), the evolution of trust might exhibit a hysteresis nature, implying a potential delay in its adjustment.

To develop assumptions about the temporal nature of trust calibration, we rely on research on mental models. Mental models are defined as “a rich and elaborate structure, reflecting the user's understanding of what the system contains, how it works, and why it works that way” (Carroll & Olson, 1987, p.12). They are built based on the driver’s experience with and resulting understanding of the automation. An unexpected automation failure creates an immediate discrepancy between the driver’s mental model and the actual functioning of the automation, thereby challenging the driver’s understanding of the system. Since humans tend not to trust what they do not understand (Wickens, 1995), the basis for the previous trust level is immediately compromised. Thus, an unexpected automation failure likely leads to an immediate and rapid decline in trust.

Compared to the trust decline, we expect that the rebuilding of trust requires a longer period of time. Before trust can be rebuilt, drivers need to perceive the automation as functioning reliably again and update their mental model to reflect the automation’s failure. The adaptation of the mental model occurs throughout the course of interaction (Beggiato & Krems, 2013) and may be considered part of the “information assimilation and belief formation” process (as described in Lee & See, 2004). Once the mental model is updated, the trust rebuilding process begins, as understanding the reason for an automation failure enhances trust (Dzindolet et al., 2003; Kraus et al., 2020). As a result, the rebuilding of trust after an automation failure is likely to take a longer period of time.

Recent research supports the assumption that there is a rapid decline in trust when an automation failure occurs, followed by a slower rebuilding process once the automation functions reliably again. In a driving setting with trust measurement after take-over requests, trust decreases but then re-establishes until the next take-over situation (Kraus et al., 2020) or the next drive (Mishler & Chen, 2023). Using frequent trust measures in the context of a remote robotic vehicle, Desai et al. (2013) descriptively showed that the trust rebuilding occurs over a longer period than the initial decline. This temporal effect was descriptively visible even though trust was only indirectly assessed (Desai et al., 2013). We expect that the temporal differences between trust decline and trust repair will become even more pronounced when using a more direct trust assessment.

Building upon research on mental models and previous empirical findings, we expect that drivers confronted with automation failures will exhibit a rapid decline in trust in the automation, and that the rebuilding of trust will occur at a slower rate once the automation works reliably again.

*Hypothesis 2a:* Trust in automation decreases immediately when automation fails, but increases over a longer period of time as it works reliably again.

### Temporary Automation Failures and Reliance Behavior Calibration

We expect that drivers’ reliance behavior calibration in response to temporary automation failures will exhibit a temporal pattern analogous to that proposed for trust calibration. As previously discussed, drivers calibrate their reliance on automation in alignment with their trust (Hoff & Bashir, 2015; Lee & See, 2004). Therefore, we expect that drivers will immediately increase their monitoring behavior when automation fails. However, as previously described, drivers need to adapt their mental model to the unexpected situation. Therefore, we expect drivers to slowly reduce their monitoring behavior over time as the automation works reliably again.

*Hypothesis 2b:* Monitoring increases immediately when automation fails, but decreases over a longer period of time as it works reliably again.

## The Impact of the Timing of Automation Failures on Trust

Previous research is inconclusive regarding the impact of the timing of automation failures on trust. Examining nonautomotive automation, Desai et al. (2012) discovered that late compared to early automation failures are more detrimental to trust, while Desai et al. (2013) found the opposite. Desai et al. (2013) concluded that the contradictory findings are attributable to the different methods used. In the initial study, Desai et al. (2012) assessed trust using a questionnaire administered after the interaction and attributed the lower trust associated with late automation failure to a recency bias. In contrast, Desai et al. (2013) computed a sum score of several surveyed trust changes within an interaction and found lower levels of trust for early automation failures.

It seems plausible that the methodology employed by Desai et al. (2013) may also have produced a biased result. The study used a sum score of trust ratings during the interaction and identified lower trust for early automation failure (Desai et al., 2013). We argue that this methodology did not allow for an adequate assessment of the full trust impairment and recovery process in cases of late automation failure, due to an insufficient number of trust measurements before the study trial ended. Therefore, the difference in the sum of all trust ratings was likely due to lower trust being measured during the trust rebuilding process for early automation failure but not for late automation failure.

Given the inconsistent findings and methodological limitations, we rely on theoretical considerations to describe the impact of the timing of automation failure. The extant literature indicates that drivers’ mental models become more accurate throughout the course of an interaction (Beggiato & Krems, 2013). Consequently, drivers who have been driving with the automation for some time possess a better understanding of its functionality. Because of their better understanding, these drivers are likely to understand automation failures better. Given that an automation failure has less of an impact on trust when drivers have an explanation for it (Dzindolet et al., 2003; Kraus et al., 2020), we expect that drivers’ trust will be less affected by an automation failure that occurs later in the automated drive, when mental models are more elaborate, than by an automation failure that occurs early in the automated drive, when mental models are still being developed.

*Hypothesis 3:* Trust in automation decreases more after an early automation failure than after a late automation failure.

## Method

### Participants

A total of 40 participants (16 females and 24 males) aged between 19 and 52 years, with a mean age of 31.75 (*SD* = 8.31), took part in the study. Participants had held their driving license on average for 13.85 years (*SD* = 8.27) and were preselected according to the following criteria: no or minimal amount of experience with automated driving and possession of a driving license for more than three years. The level of pre-experience was equally distributed between the experimental groups (10 and 11 participants without any pre-experience). Our research complied with the American Psychological Association Code of Ethics and informed consent was obtained from each participant.

### Experimental Design and Procedure

Each participant drove within three experimental conditions (a combination of within and between subject design). The first two drives differed in the risk level present during the drive (within-subject: *low-risk drive* and *high-risk drive*). The third drive was either with automation failure at the beginning or the end of the drive (between-subject: *early-failure drive* or *late-failure drive*).

After an introduction, participants completed a familiarization drive. Each experimental drive lasted approximately 15 min, followed by a short break that included questionnaires. Participants did not receive information about system limitations as the aim was to test drivers’ ad hoc ability to adapt to changing driving contexts. Drivers were instructed that they could perform NDRAs while using driving automation but remain responsible for driving safety.

All drives consisted of the same 33 km long highway segment. Random cars were driving in the right lane at a velocity below the speed limit of 130 km/h, thus requiring participants to use the left lane.

In the *low-risk drive*, the weather was sunny and the automated driving system was highly reliable. The *high-risk drive* was operationalized by combining environmental factors (heavy rain and low visibility), slight swerves towards the lane marking (at 4.33 km, 9.39 km, 16.61 km, and 25.28 km; see Note for an illustration), and a monetary incentive to introduce actual risk for the participants in the simulated driving situation. The environmental manipulation of bad weather conditions was retrieved from Li et al. (2019), who compared different risk situations in a driving simulation and found it to be effective in inducing situational risk.

Vehicle swerving towards the lane marking was implemented to meet the risk definition of an increased likelihood of a negative event. Swerving towards the lane marking is not a critical event in itself but rather an indication that such an event could occur, for example, a swerve beyond one’s own lane that is likely to result in an accident.

**Figure 1.**
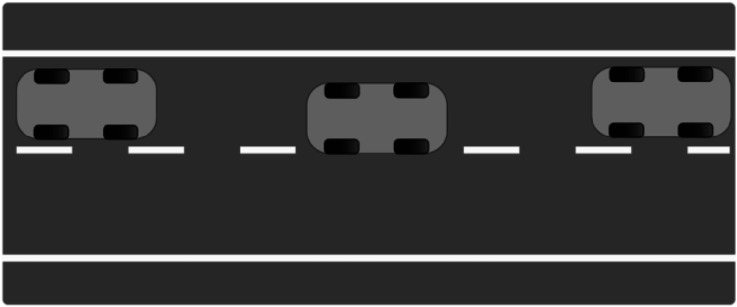
Illustration of the slight swerve towards the lane marking. Slight swerves towards the lane marking were part of the *high-risk drive* (the car drives from the left to the right).

The monetary incentive was implemented to compensate for the missing vulnerability in a simulation setting as is often done in simulation studies (e.g., Desai et al., 2013; Desai et al., 2012; Dzindolet et al., 2003; Lee & Moray, 1992, 1994). The incentive was presented in terms of a loss: half of the originally advertised compensation was presented as contingent on the overall driving performance in the *high-risk drive*. After completing the experiment, all participants received the full compensation (Figure 1).

**Figure 2.**
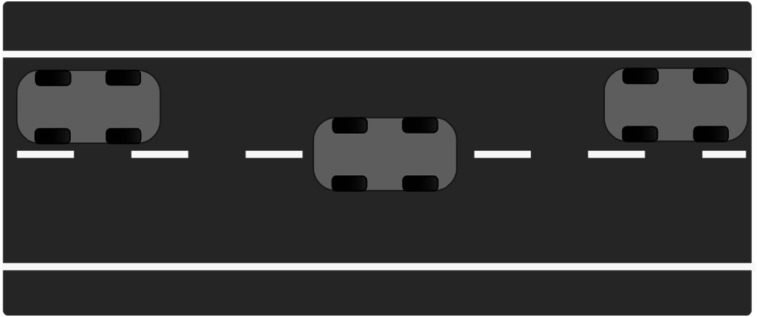
Illustration of the strong swerve. Strong swerves on to the neighbor lane were used as automation system failure in the *early-failure drive* and *late-failure drive* (the car drives from left to right).

The automation failure in the *early-failure drive* and *late-failure drive* was implemented as strong swerves to the middle lane for the duration of approximately 20 s, either at 4.33 km and 9.39 km (*early-failure drive*) or at 21.61 km and 26.67 km (*late-failure drive;* for an illustration see Figure 2). Situational risk was not manipulated for these drives.

### Equipment and Data Acquisition

#### Driving Simulator

The study was conducted using a semi-static driving simulator equipped with an automated driving system that keeps the lane and the set speed. Within the driving scenario used, the simulator’s driving automation fulfilled the requirements for conditional driving automation as defined by SAE International (2021).

#### NDRA

The online video platform YouTube, presented on a 10.1-inch Android tablet, was used as NDRA. We used this task because watching videos is a realistic activity a driver could choose while using an automated vehicle.

### Dependent Variables

#### Trust in Automation

**Figure 3.**
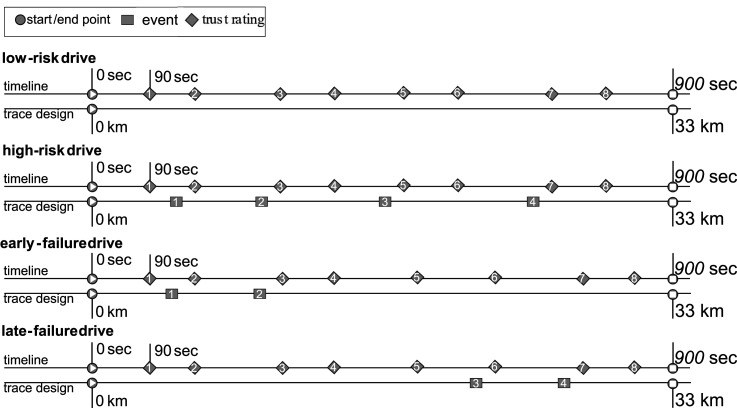
Timing of the events and trust ratings in the four conditions. Trust ratings were always surveyed 10 and 90 s after the events. In the *low-risk drive* timing was based on the estimated average timing of events in the *high-risk drive*. While the *high-risk-drive* included a threefold risk implementation with slight swerving events, the early- and late-failure drives included strong swerves as automation failures without any other manipulation.

Trust was assessed eight times during each drive using the single item “How much do you trust the system at the moment? (from 1 = not at all, to 7 = completely).” It was presented and answered verbally using the simulator’s intercommunication system. The timing of the items was defined by the total time since the participant started driving and the time since the end of the last automation failure (see Figure 3). A mean value of all ratings per drive was calculated as an overall trust score throughout the interaction. While single-item measures are always a topic of debate (e.g., Matthews et al., 2022), we used this approach in accordance with previous automated driving studies (e.g., Ebinger et al., 2024; Hergeth et al., 2016; Körber, Prasch, & Bengler, 2018), as it allows for repeated measurements and a direct temporal representation (Yang et al., 2017).

#### System Use

Participants could disengage the automation system whenever they deemed it necessary. The number of disengagements was automatically recorded.

#### Monitoring Behavior

Participants wore mobile eye-tracking glasses (Dikablis Glasses 3) to record their monitoring behavior. Results are reported regarding the monitoring ratio (percentage of time looking at the NDRA or driving task).

## Results

We applied the Bonferroni correction in cases of multiple comparisons (p* denotes Bonferroni-corrected *p*-values). If the resulting value was greater than 1, it is reported as 1. T-tests were conducted despite partial violations of normality, as simulation studies have indicated that they are robust against such violations (Wilcox, 2012). In cases of nonhomogeneous variances, the Welch t-test was used.

### Risk, Trust in Automation and Reliance Behavior

To test whether trust in automation is lower when driving in a high risk situation compared to a low risk situation, we computed a t-test for dependent measures. In support of *hypothesis 1a*, we found that the average level of trust during the interaction is significantly lower for the *high-risk drive* (*M* = 3.77, *SD* = 1.19) than for the *low-risk drive* (*M* = 5.72, *SD* = .98; *t* (39) = 10.40, p* < .001). Cohen’s (1988) effect size value (*d*_*z*_ = 1.78) indicates a large effect.

To test whether driving automation is used less in a high risk situation compared to a low risk situation, we computed a t-test for dependent measures. In support of *hypothesis 1b*, we found that the average number of manual takeovers is significantly higher for the *high-risk drive* (*M* = 3.32, *SD* = 2.01) than for the *low-risk drive* (*M* = 1.32, *SD* = 1.25; *t* (33) = −4.93, p* <.001). Cohen’s (1988) effect size value (*d*_*z*_ = −1.13) indicates a large effect.

To test whether monitoring of the driving task is higher when driving in a high risk situation compared to a low risk situation, we computed a t-test for dependent measures. In support of *hypothesis 1c*, we found that the average monitoring ratio on the NDRA (compared to the driving task, %) is significantly lower for the *high-risk drive* (*M* = 27.40, *SD* = 18.76) than for the *low-risk drive* (*M* = 42.46, *SD* = 23.03; *t* (32) = 5.76, p* <.001). Cohen’s (1988) effect size value (*d*_*z*_ = .72) indicates a large effect.

### Trust in Automation and Monitoring After Automation Failure

To test whether trust in automation decreases over a short period of time when automation fails but increases over a longer period of time when the system works reliably again, we computed a repeated measures ANOVA followed by pairwise comparisons using dependent t-tests. We conducted t-tests between the first and last measurement, as well as between subsequent measurements. The comparisons were stopped once trust did not significantly change anymore from one measurement to the next.

**Figure 4.**
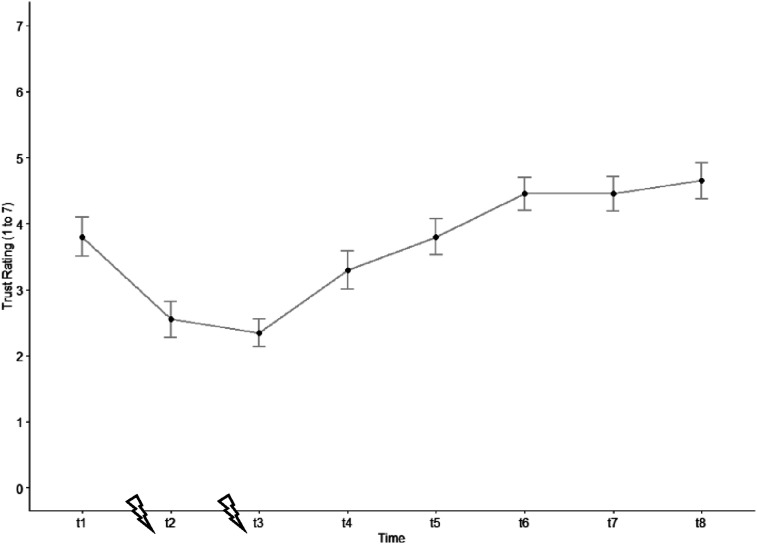
Trust evolution (early-failure drive). The blizzard symbol indicates the automation failures and the trust measurement times (t1 to t8) are specified in figure 3.

The ANOVA revealed a significant main effect of the factor time (*F* (3.79, 71.97) = 27.00, *p* < .001, η^2^_G_ = .33). In support of *hypothesis 2a*, we found that trust in automation significantly decreased after the first automation failure. Already 10 s (t4) after the end of the second automation failure the level of trust significantly increased again and kept increasing until t6 (for descriptive statistics see Figure 4; for test statistics see Table 1).

**Table 1.** Test statistics for the pairwise trust comparisons.

Time	*t*-value	*p*	p*	*d*
1 vs. 8	−3.66	.002	.014	−.82
1 vs. 2	4.63	.000	.001	1.03
2 vs. 3	.78	.447	1	.17
3 vs. 4	−3.87	.001	.007	−.86
4 vs. 5	−4.36	.000	.002	−.98
5 vs. 6	−4.95	.000	.001	−1.11
6 vs. 7	0.00	1	1	0

*Note.* For all groups *N* = 20; Asterisk indicates Bonferroni corrected p-value.

To test whether monitoring of the driving task increases over a short period of time when automation fails but decreases over a longer period of time when the system works reliably again, we analyzed monitoring by using 1-min time periods centered on the onset of the first automation failure. We followed the same analysis procedure using repeated measures ANOVA and dependent t-tests as for *hypotheses 2a*.

**Figure 5.**
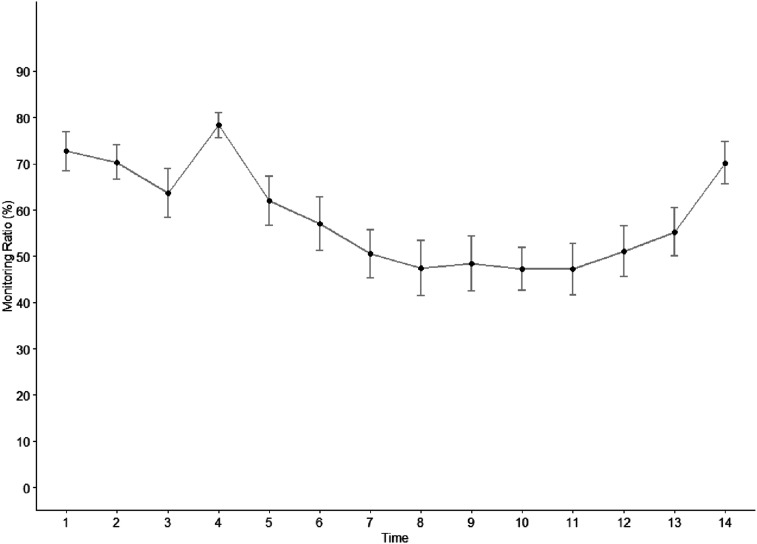
Monitoring evolution (early-failure drive). Automation failures cannot be indicated as the 1-min time bins are not aligned with each automation failure as it is the case for the trust ratings.

The ANOVA revealed a significant main effect of the factor time *F* (5.24, 94.25) = 9.123, *p* < .001, η^2^_G_ = .191. However, contrary to hypothesis 2b, the pairwise comparisons indicate no temporal difference in how drivers increase their monitoring in case of automation failure and decrease it as it works reliably again. The monitoring of the driving task significantly increased after the failure and then, again, decreased. There is no significant difference in monitoring between the beginning and the end of the interaction (for descriptive statistics, see Figure 5; for test statistics, see Table 2).

**Table 2.** Test statistics for the pairwise monitoring comparisons.

Time	*t*-value	*p*	p*	d
1 vs. 14	.51	.616	1	.12
1 vs. 2	.53	.601	1	.12
2 vs. 3	1.61	.125	.750	.37
3 vs. 4	−3.20	.005	.030	−.74
4 vs. 5	4.95	.000	.000	1.14
5 vs. 6	1.36	.192	1	.31

*Note.* For all groups, *N* = 19; Asterisk indicates Bonferroni corrected p-value.

### Timing of Automation Failure and Trust in Automation

To test whether trust in automation decreased more strongly after an early than after a late automation failure, we used the relative trust change from the rating before to the rating after the automation failure. We used this method as our trust evolution analysis shows that trust builds up over time. Thus, using the absolute value could have resulted in a biased finding, as late in interaction the absolute potential for trust decrease is higher due to the higher trust. By taking the pre-failure trust rating as a baseline (=100%) and analyzing the relative change to the postfailure trust score we mitigated this potential bias.

Contrary to the hypothesis, a Welch two sample t-test for independent measures indicates that there is no significant difference in the average percentual trust decrement after an early (*early-failure drive*; *M* = 30.92, *SD* = 27.40) or a late automation failure (*late-failure drive*; *M* = 34.64, *SD* = 20.50; *t* (35.19) = -.50, *p* = .629).

## Discussion

With conditional driving automation, drivers become fallback-ready users who need to calibrate their trust and reliance in response to risk and automation failure. In the present study, when confronted with high risk (as compared to low risk), drivers reported lower trust, used the automation less, and monitored the driving task more intensely. When faced with automation failures, drivers immediately trusted less and monitored more, but recalibrated as the automation worked reliably again. For trust, the rebuilding continued longer than the decline, but there was no such difference for monitoring behavior. Contrary to previous findings (Desai et al., 2012, 2013), the relative trust decrement did not differ between early and late automation failures.

### Theoretical Implications

#### Trust and Reliance in Situations with High Risk

The results show that drivers not only adapt their level of trust (Li et al., 2019), but also their reliance behavior in response to risk. Despite the possibility of performing NDRA when using conditional driving automation, drivers are aware of the driving environment and the automation’s behavior. The concurrent adaptation of trust and reliance behavior is in line with the assumed trust-reliance feedback loop (Hoff & Bashir, 2015; Lee & See, 2004). Potential contextual factors that could have caused discrepancies between the attitude trust and actual behavior did not occur. In addition, the two subcomponents of reliance, monitoring, and automation use were equally adapted to the situation.

#### Trust Calibration After Automation Failure

The calibration process of trust and reliance in case of automation failure shown in this study follows the trust-reliance feedback loop process outlined in various trust models (Hoff & Bashir, 2015; Lee & See, 2004). As the trust-reliance calibration feedback loop suggests, trust and reliance were continuously re-calibrated in a congruent way. Drivers trust the automation less and rely less on it when confronted with automation failure and increase trust and reliance as the automation works reliably again. Mishler and Chen (2023) found trust rebuilding across drives and Kraus et al. (2020) observed it across take-over situations. Our finding extends these insights by showing the dynamic trust calibration process in greater temporal detail. The ongoing adaptations over time indicate a high temporal specificity of drivers’ trust which Lee and See (2004) describe as an indicator of calibration quality.

Our study results support the assumption that in situations with temporary automation failures trust and reliance decline immediately and, as the automation works reliably, build up again. Hereby the increases in trust continued over a longer period of time. The concept of mental models explains the immediate trust reduction and its gradual rebuilding over time. Inadequate mental models can reduce trust in automation (Wickens, 1995). Further, in the context of human–robot teams, it has already been suggested that the mental model can be essential in the process of calibrating trust (Visser et al., 2020). An unexpected automation failure can cause a mismatch between the mental model and the actual behavior of the automation, resulting in an immediate reduction of trust. The fact that the unexpected automation failure must be integrated into the mental model explains why trust rebuilding takes longer than its decrement.

Integrating the mental model process into the trust-reliance calibration feedback loop would allow for a better explanation of trust calibration, including its temporal dimension. We propose extending the “information assimilation” process described by Lee and See (2004) to include an explanation of the mental model processes. Our findings suggest that this process involves comparing the mental model to the perceived system behavior. Future studies should include a detailed assessment of the mental model to better understand the cognitive processes.

Contrary to our expectations, drivers’ monitoring increased after the automation failure as quickly as it decreased in response to the failure. While monitoring continued to increase descriptively, there were no further significant changes. The study data do not allow us to conclude whether this is the result of a less gradual adaptation of the monitoring or the result of an actual difference in trust and behavioral calibration.

#### Timing of Automation Failure

As we did not find any timing of automation failure effect, our theoretical explanation that late failures have a stronger impact on trust because an already established mental model (which is still less elaborated in case of an early failure) has to be restructured, seems inadequate. Although the drivers had longer interaction time with the automation, their reactions to an automation failure did not differ from the reactions of drivers who experienced an early failure. This suggests that, despite likely having more accurate mental models, the unexpected unreliability of the system led to a similar loss of trust. Future studies should compare the effects of automation failures on the temporal dynamics of trust and reliance behavior between drivers who are aware versus unaware of potential automation failures.

#### Measurement of Trust in Automation

The measurement of trust in automation using a single-item measure is a controversial topic (e.g., discussion in Matthews et al., 2022). The negative aspects include the low precision of single-item measures (Fuchs & Diamantopoulos, 2009) and the lack of information about the underlying reasons for the trust score (Körber, Baseler, & Bengler, 2018). Matthews et al. (2022) differentiate whether a construct is multi-dimensional and thus requires multiple items to ensure a participant responds to all its aspects.

For a study like ours, a single-item measure of trust is effective. In our reference studies, some paused the simulation for trust scales (as done in Kraus et al., 2020), while in other studies participants only indicated a plus or minus in their trust while interacting with the automation (Desai et al., 2013). Matthews et al. (2022) demonstrated the effectiveness of single-item measures across several constructs. Focusing specifically on trust in automation, several automated driving studies used single-trust items (e.g., Ebinger et al., 2024; Hergeth et al., 2016; Körber, Prasch, & Bengler, 2018). Yang et al. (2017) suggest that several single-item measures better represent an interaction than a retrospective trust scale. In our study, the single-item was timed event-based and allowed a highly temporally specific trust measurement throughout driving.

### Practical Implications

Our study shows the need for supporting the re-calibration process. Drivers’ trust in automation and reliance behavior decreases immediately after an automation failure, and trust only gradually rebuilds when the system is operating reliably again. In the case of a temporary failure, as in our study scenario, the drivers’ reluctance to use the system results in a loss of the benefits of driving automation for some time. Drivers do not perform the NDRAs and do not benefit from the automation’s safety benefits during the re-calibration process. Several studies explored methods such as information displays (e.g., Dzindolet et al., 2003) or tutoring systems (e.g., Neuhuber et al., 2022) that could be used to support the re-calibration process.

In addition, drivers would benefit from NDRAs that are designed with the dynamics of reliance in mind. In our study with YouTube videos as NDRAs, the driver had to manually pause the video either before or after reacting to the situation. We did not measure this as a variable, but we could clearly observe that this aspect added complexity to the driver’s process of noticing the error and feeling the urge to react to it. Therefore, an NDRA that automatically pauses in such a situation would allow the drivers to more easily redirect their attention and not rely solely on the automation.

### Limitations

Regarding limitations, the within-subjects study design must be discussed critically. All participants completed first the low-risk drive and then the high-risk drive. The drives were not counterbalanced due to other research questions addressed by collaborating researchers. However, it is not expected that this impacted the validity of the findings, as any potential bias would have been against the hypotheses. Recent research showed that trust and reliance toward automation increase while using it (e.g., Hergeth et al., 2017), thus a position effect would have increased the trust and reliance behavior towards the automation in the second drive. However, the findings showed lower trust and reliance for the second drive due to the high risk condition.

After the within-subject risk drives, the participants were split into two groups for the third drive with either early or late automation failure. Despite breaks between each drive, it could be argued that the participants experienced the three drives as one long interaction. In this case, the early failure would more likely be experienced as occurring in the middle. To counteract this and maintain a clear distinction between the different experimental conditions, we explicitly informed participants that they were using different versions of the automation system for each drive.

### Conclusion

We aimed to examine the impact of risk and automation failure on drivers’ trust and reliance calibration. Our results indicate that drivers calibrate trust and reliance to risk. In a high-risk situation, drivers reported lower trust, took over manual control more often, and monitor more than in a low-risk situation. Following automation failure, drivers decreased their trust and monitoring immediately. As the automation works reliable again, the increase in trust persisted over a longer period of time than its previous decrease. Monitoring did not show a continued increase. The timing of automation failure had no impact on the trust decline. Our results suggest incorporating mental model processes into the theoretically described trust-reliance calibration feedback loop.

## Key Points

- Trust and reliance in conditional driving automation decrease under high-risk driving conditions
- After being confronted with an automation failure, trust and reliance immediately decrease but fully recover again
- The timing of automation failure does not influence its impact on trust
